# Manifestation of morphea in a patient with myasthenia gravis under therapy with zilucoplan

**DOI:** 10.1111/ddg.15919

**Published:** 2025-10-07

**Authors:** Phoebe Wellmann, Jana Zschüntzsch, Michael P. Schön, Rotraut Mössner

**Affiliations:** ^1^ Department of Dermatology Venereology and Allergology University Medical Center Göttingen Göttingen Germany; ^2^ Department of Neurology University Medical Center Göttingen Göttingen Germany

Dear Editors,

Zilucoplan, a subcutaneously administered inhibitor of complement factor C5, was approved by the *European* *Commission* in December 2023 for the treatment of adult patients with anti‐acetylcholine receptor antibody‐positive generalized myasthenia gravis (MG).^1^ It is a synthetic macrocyclic peptide of 15 amino acids.^2^ In clinical trials, zilucoplan was generally well‐tolerated, with common side effects including injection site reactions, upper respiratory tract infections, diarrhea, and morphea.^3^, ^4^

A 36‐year‐old woman had insufficiently controlled MG symptoms under systemic corticosteroids and recurrent intravenous immunoglobulin cycles. Previous therapies with methylprednisolone, azathioprine, mycophenolate mofetil and methotrexate were discontinued due to insufficient clinical response or adverse events. Her *Myasthenia Gravis Foundation of America (MGFA) classification score* was IIIa. She was treated in the RAISE clinical trial (MG0010),^3^ and its extension, RAISE‐XT (MG0011),^5^ investigating zilucoplan therapy in MG (September 2021–March 2024). The patient continued commercial zilucoplan use after having completed the studies. After 2.5 years of effective zilucoplan treatment measured by an improvement in MG‐ADL (activity of daily living) from 10 at screening to 0 points, she presented with hyperpigmented and sclerotic patches on the lateral aspects of the breasts, submammary areas, inguinal regions and a 4–5 cm sclerotic macule on the left shoulder (Figure 1). These lesions had been present for approximately one year. Additionally, she reported occasional limb pain and episodic cold‐induced bluish discoloration, stiffness, and itching of the fingers.

**FIGURE 1:**
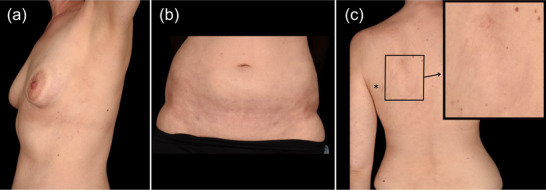
Clinical images of morphea at initial presentation. (a) Hyperpigmented, sclerotic patches, semicircular in shape, on the left lateral aspect of the breast with a clear periareolar margin. (b) Hyperpigmented, sclerotic inguinal patches. (c) 4–5 cm sclerotic macule on the left shoulder. The asterisk marks a scar from an excision years ago.

A skin biopsy from the left shoulder revealed features consistent with morphea: mild basal hyperpigmentation, compact orthokeratosis, homogenized collagenous tissue in the deep dermis, and mild perivascular lymphocytic infiltration with occasional plasma cells. Autoimmune diagnostics showed positive ANA titers (granular nucleoplasm 1:1,000, cytoplasm‐positive 1:320), elevated SSA antibodies > 240.0 U/ml and positive Pm‐Scl‐75 antibodies. Common systemic sclerosis antibodies, including SCL‐70, CENP‐B, RNA‐polymerase‐III and fibrillarin, were negative. Mouth dryness was reported upon inquiry. A lower lip biopsy was unremarkable and finger capillaroscopy was nonspecific. Plaque‐type circumscribed morphea was diagnosed. Skin lesions were treated with highly potent glucocorticoids and ultraviolet (UV)A1 phototherapy. Phototherapy was discontinued after 9 weeks due to lack of improvement, while topical corticosteroid treatment was continued. A systemic therapy with methotrexate, as recommended as first‐line treatment for morphea in current guidelines, was not given, as the patient had previously received methotrexate for 3 months to treat myasthenia gravis but had to discontinue the treatment due to adverse events including nausea, vomiting, pain and flu‐like symptoms.^6^ Despite morphea manifestation, the patient initially chose to continue zilucoplan because of the excellent improvement of her MG symptoms.

Morphea, also referred to as localized scleroderma, is a rare inflammatory connective tissue disease primarily affecting the skin, though it can also involve fascia, muscles, bones, and the central nervous system. Plaque‐type morphea, the most common subtype, is characterized by erythematous sclerotic lesions with a livid border in the active stage, progressing to central sclerosis and potentially becoming atrophic with hypo‐ or hyperpigmentation.^6^, ^7^ Treatment options include topical therapies, UV therapies, systemic therapies (such as methotrexate and glucocorticoids) and physiotherapy.^6^ Potential trigger factors of morphea include infections, mechanical trauma, radiation therapy and drugs such as bleomycin, D‐penicillamine, vitamin K1, L‐5‐hydroxytryptophan plus carbidopa, and balicatib.^8^ According to the *Summary of Product Characteristics* (SPC),^4^ morphea is also a common side effect of zilucoplan. It was observed as an adverse drug reaction in the long‐term open label extension study MG0011. For this study, 34 patients (17%) and 166 patients (83%) were enrolled from the prior double‐blind, pivotal phase II and phase III MG0009 and MG0010 studies, respectively. Notably, morphea was not reported in the interim analysis of the RAISE‐XT study conducted by Howard et al., as the cutoff date for data collection was September 2022. At the cutoff date, the median exposure to zilucoplan was 1.2 years, with a cumulative exposure of 321.4 patient‐years.^5^ Morphea cases were identified only after this date, when additional data had been collected. Most morphea cases occurred after more than one year on zilucoplan, were mild to moderate and did not require discontinuation of zilucoplan.^4^ In our patient, zilucoplan was discontinued after 32 months, as skin lesions slowly worsened despite ongoing topical corticosteroid treatment. After discontinuation of zilucoplan and continuation of topical therapy, morphea remained unchanged. However, MG progressed, so that therapy with an inhibitor of the neonatal Fc receptor was started.

The pathomechanism of morphea is not well understood. It is considered an autoimmune disease initially characterized by T‐cell‐driven skin inflammation around blood vessels and skin adnexa. Early stages involve the recruitment of proinflammatory and fibroblast‐activating TH1 and TH17 cells, while progression leads to a shift to TH2 cytokines and further fibrosis.^6^ Venneker et al. suggested that reduced expression of complement regulatory molecules in the endothelium may contribute to vascular damage and fibrosis.^9^

While a coincidental manifestation of morphea independent of zilucoplan cannot be ruled out, the SPC lists morphea as a common side effect defined as occurring in > 1/100 and < 1/10 of patients.^4^ In contrast, the incidence of morphea is reported to be 4 to 27 per one million individuals in the general population.^6^, ^7^ There are limited data on the incidence of morphea in the MG population, but it seems to be higher in patients with autoimmune conditions compared to the general population.^10^

To the best of our knowledge, morphea induced by other complement 5 inhibitors has not been reported. It remains to be investigated whether this is a substance specific effect or an off‐target pharmacological effect, although there is currently no evidence for a class effect. Further studies are needed to investigate the pathomechanism of morphea induced by zilucoplan and optimize its management.

## CONFLICT OF INTEREST STATEMENT

P.W. reports no conflict of interest. J.Z. has been an advisor and/or received speakers’ honoraria and/or received grants and/or participated in clinical trials of the following companies: Alexion, Amicus, Argenx, Kedrion, Roche, Sanofi, UCB. M.P.S. has been an advisor and/or received speakers’ honoraria and/or received grants and/or participated in clinical trials of the following companies: AbbVie, Almirall, Biogen, Boehringer‐Ingelheim, Janssen‐Cilag, Leo, Lilly, Novartis, UCB. R.M. has been an advisor and/or received speakers’ honoraria and/or received grants and/or participated in clinical trials of the following companies: AbbVie, Almirall, Biogen, Boehringer‐Ingelheim, Celgene, Janssen‐Cilag, Leo Pharma, Lilly, MSD Sharp & Dohme, Novartis, Pfizer, UCB.
